# Students' and lecturers' perspectives on the implementation of online learning in medical education due to COVID-19 in Germany: a cross-sectional pilot study

**DOI:** 10.3389/fmed.2023.1145651

**Published:** 2023-04-24

**Authors:** Stefan F. Hertling, David A. Back, Mario Kaiser, Franziska M. Loos, Ekkehard Schleußner, Isabel Graul

**Affiliations:** ^1^Department of Obstetrics and Gynaecology, University Hospital Jena, Jena, Germany; ^2^Orthopedic Department, University Hospital Jena, Eisenberg, Germany; ^3^Clinic for Traumatology and Orthopedics, Bundeswehr Hospital Berlin, Berlin, Germany; ^4^Dieter Scheffner Center for Medical Education and Educational Research, Charité–Universitätsmedizin Berlin, Berlin, Germany; ^5^Modul Integration Optics, Jenoptik Light and Optics Devision, Jena, Germany; ^6^Practice for Orthopedics and Shoulder Surgery Leipzig, Leipzig, Germany; ^7^Department of Trauma, Hand and Reconstructive Surgery, Jena University Hospital, Friedrich Schiller University Jena, Halle (Saale), Germany; ^8^Department of Orthopedic and Trauma Surgery, Martin-Luther University Halle-Wittenberg, Halle (Saale), Germany

**Keywords:** digital teaching, virtual teaching, sports medicine, e-learning, medical education, COVID

## Abstract

**Background:**

During the coronavirus disease 2019 (COVID-19) crisis, many things changed in universities around the world. In-person learning was not possible. Instead, courses were offered in digital form. The sudden change posed enormous challenges to universities, students, and teachers. The aim of this study was to investigate the disadvantages as well as the advantages and opportunities of digital learning.

**Objective:**

This study investigated the evaluation of an elective module by medical students and teachers in the traditional in-person and virtual teaching forms during the COVID-19 pandemic.

**Methods:**

Using the elective module “Sports Medicine,” which includes both lectures and practical units, the opinions of the medical students about conventional teaching compared to digital instruction were evaluated. In the winter semester of 2019/2020, all classes were taught face-to-face but had to be switched to virtual teaching in the summer semester of 2020 on an *ad hoc* basis due to the pandemic. The students were asked to answer questions on general conditions, participant behavior, instructor evaluation, skill acquisition, topic selection, and overall evaluation after both forms of teaching. Likewise, the lecturers of both courses were queried in semiqualitative interviews about the same topics. Descriptive data analysis was performed to process the data.

**Results:**

The students perceived digital teaching to be superior in most subareas compared to in-person teaching in terms of framework, instructor evaluation, skill acquisition, topic selection, and overall rating. Medical students seemed to feel better with digital teaching in most areas of evaluation. The lecturers found the new form of teaching rather unsettling and criticized the lack of verbal and especially nonverbal communication as well as the short preparation time for the new challenge. The instructors were uncomfortable with some aspects of the virtual teaching format.

**Conclusion:**

In the wake of the COVID-19 pandemic, medical schools should rapidly digitize their teaching offerings and support faculty members in their computer-based competence with continuing education opportunities and time resources.

## Introduction

Severe acute respiratory syndrome coronavirus 2 (SARS-CoV-2), which caused COVID-19, is one of the most aggressive and deadly infectious diseases (1). COVID-19 was declared a pandemic by the WHO on 30 January 2020 (2). Therefore, most countries implemented physical distancing measures to decelerate the infection rate (3–5). The pandemic dramatically disrupted many areas of life. Teaching was digitized in most medical schools to prevent the rotation of students between departments and hospitals, which might cause them to become potential vectors in the transmission of the disease (6). Previous formats for imparting knowledge such as lectures, seminars, group lessons, and practical exercises have to be terminated and abruptly converted into online-based variants (7). This change required consideration from both sides, the teachers and the students. In a very short time, the whole world was dealing with the challenge of maintaining high-quality education through new digital platforms. There have been no historical records of such a vast sudden shift toward e-learning (8). It has long been acknowledged that online instructional methods are efficient tools for learning (9). However, aspects such as limited nonverbal communication, limited interaction between students and professors, accessibility of materials, and time management were also discussed as critical influencing factors of the students' opinions on online courses (10). The main difference between traditional and online-learning sessions is that the latter allows students to learn from their preferred locations (11, 12). Technological solutions allow lessons to be delivered to groups and real-time processing of individual student responses. Online meeting tools like virtual web-based platforms such as Zoom^®^, GoToMeeting, WebEx Meeting, Adobe Connect, and many more can be used for providing instructions. They also allow synchronous sessions and multiple users to participate at the same time (13). The instructor of the session must plan for the learning curve required for students to use virtual web-based platforms effectively and not assume that all students have the necessary practical knowledge to use them. In addition, students may engage in other activities during the lecture and not actively participate. However, there are concerns that practical medical content will be rather poorly delivered through digital forms of instruction (14, 15). The fruitful use of technology in medical education depends on the willingness and expertise of the faculty to use this technology to facilitate learning. Training physicians with these skills requires a break from traditional teaching methodology (16). Therefore, it seems that training in the mastery of computer technology is a neglected skill among faculties that should be mandatory for the improvement of medical universities. An organized and clear institutional approach is needed to formulate a well-regulated and efficient system that can facilitate the adoption of structured methods by faculty members during the implementation of an online-learning module (17).

The aim of this study was to evaluate the students' and lecturers' perspectives on digital and conventional teaching of a well-established elective module at a mid-sized multicultural German medical school.

## Methods

### Study setting

This mixed-methods study took place at the medical school of a German University hospital in the context of the quick change from traditional teaching to digital teaching due to the COVID-19 pandemic, which was realized in 2020. At the time of the study, approximately 2090 students were enrolled at the medical school. Sample calculation was not carried out but was defined based on the course participants. Digital teaching in medical studies has not been regularly practiced there before. During clinical semesters, undergraduate students in the third to the fifth year of training must choose elective modules from a variety of course programs covering different medical fields that are usually not covered by the mandatory curriculum. The modules are in direct competition with the students' interests and comprise 28 teaching hours (45 min each). Each student must attend at least 80 lectures in an elective subject. Only the most-selected modules by the students were taught. Due to the COVID-19 pandemic, lectures had to be converted into a digital format in a very short time for them to take place. Some lectures could not be completely digitized and many had to be canceled. In the elective module “Sports Medicine,” 14 events of 90 min each were held by three lecturers in person. After conversion to an internet web-based platform, the lectures took place in a slimmed-down version with eight events of 45 min with the same three lecturers. The elective is a multifaceted teaching format consisting of practical applications, case studies, and demonstration of examination methods from the field of sports medicine.

### Study design

A mixed-methods approach was chosen that consists of an abductive qualitative study based on semi-structured interviews and a cross-sectional study using course evaluation questionnaires at the end of the course. An abductive analysis of the interview transcripts and the results of the open-ended questions of the faculty questionnaire was conducted to identify the predominant themes (18). The predefined themes included teaching formats and learning objectives. Qualitative data included interview transcripts and results of the open-ended questionnaires. For the qualitative interviews, we did not test whether saturation was achieved because only 3 interviewees participated in digital and face-to-face instructions. Items from the questionnaire with a five-point Likert scale as the response format were considered quantitative data. The use of the mixed method was deliberately chosen by the authors for this study so that the qualitative and quantitative research approaches can be combined to address the specific research interest, namely the influence of digital teaching during the COVID-19 pandemic in direct comparison to face-to-face teaching in medical higher education with complex, practice-oriented teaching in the clinical section. The mixed methods approach allows our research question to be viewed from different perspectives (students and lecturers).

### Ethical approval and consent to participate

The ethical approval for this study was granted by the local ethics committee of the University of Jena (2019–1456–Bef). Participation in the study was voluntary. Informed consent for the study was obtained by voluntarily submitting the questionnaire. Written informed consent was obtained regarding the voluntary nature of participation and all data were collected anonymously. Minor participants were not represented in the cohort. The ethics committee explicitly waived the requirement for written informed consent from participants.

### Inclusion and exclusion criteria

The inclusion criteria for the students were as follows: must be over the age of 18 years, must be a student of Human Medicine at the University of Jena in the clinical section, and must participate in the elective subject tutorial Sports Medicine. The inclusion criteria for the lecturers were as follows: must be over the age of 18 years and must be a lecturer of Human Medicine at the University of Jena for the elective subject tutorial Sports Medicine.

### Data collection

Between December 2020 and February 2021, semi-structured interviews with three lecturers of both face-to-face and digital lessons in the elective module “sports medicine” were conducted. We developed the interview guidelines according to the questions (Table 1) and tested and adjusted the questions during pilot interviews within the research team. The interviews were conducted in German *via* phone calls and were recorded and transcribed by the interviewer. The median interview length was 19 min (range 11–28 min). The questions were asked in German.

**Table 1:** Questions of the structured interview.

How did you feel about the framework of both face-to-face and digital instruction formats?
How did you find the contact with the students during the lecture?
How did you feel about the implementation of your own competencies as a lecturer in the lecture formats?
How did you feel about the knowledge transfer and prior knowledge of the students?
How did you feel about the two lecture formats as a lecturer?

### Students' evaluation

The standardized student evaluation takes place at the end of the elective subject tutorial sports medicine. The student evaluation questionnaire was given to the students on the last day of the module as a paper version for the in-person lessons (*n* = 25) in January of the winter semester 2019/2020, i.e., before the pandemic and digitally for the virtual lessons (*n* = 26) in July of the summer semester 2020, i.e., during the pandemic. No incentive or compensation was given to the survey participants. There was a difference in the number of students who attended the face-to-face and digital format lectures. This was due to logistical circumstances. Before the start of the respective elective, the maximum number of participants is determined by the medical faculty. For the in-person attendance phase, the maximum number of students was 25 and for the digital format, the maximum number of students was 26. The evaluation form included 29 questions using a Likert scale (1 = strongly disagree, 2 = disagree, 3 = undecided, 4 = agree, and 5 = strongly agree) regarding the framework conditions of the lessons (5 questions), the participants and their behavior (3 questions), the lecturers and their structure of the lecture (6 questions), the qualification, which could be imparted by the lecture (7 questions), the interest of the students and their assessment of the rate of referrals and existing knowledge (4 questions), and the overall assessment of satisfaction with the lecture (4 questions). Furthermore, the students were asked about some epidemiological data, the time required for preparation, the expected amount of work, and the amount of work performed. A place for open comments about good and bad findings at the lectures was provided. The questions were asked in German.

### Data analysis

We performed an abductive analysis of the interview transcripts and the results of the open-ended questions from the lecturers and students to identify the predominant themes. Descriptive data analysis of the questionnaire items was conducted using Microsoft Excel 2020 (version 16.35). The *P*-values were calculated using the Mann–Whitney U test with the Statistical Package for the Social Sciences, SPSS (version 17.0, SPSS Inc. Chicago, IL, USA). A *P*-value of < 0.05 was considered significant.

### Response-bias

To reduce bias errors, the research design was constructed in such a way that the respondents are not exposed to intentional or unintentional misrepresentations. Due to the maximum anonymity of the survey, the questionnaires were answered without personal contact with the lecturers as the questionnaires were distributed and collected again by independent members of the medical faculty (questionnaires on paper) or the questionnaires were digitally distributed directly to the students (online). The faculty interviews were conducted by an independent person who is also a member of the medical faculty. The study participants had enough time to fill in the questionnaires. The questionnaire for the students was previously tested on a small group of 10 students. The questions were then modified. The questions for the lecturers were created according to a standardized scheme (see Methods section). The possible number of lecturers is only three as only they were allowed to teach the elective subject. All three lecturers participated in the interview.

## Results

We constructed this mixed-methods study based on interviews with three lecturers of sports medicine and a student evaluation questionnaire of digital and face-to-face lessons.

### Quantitative results

**Figure 1:**
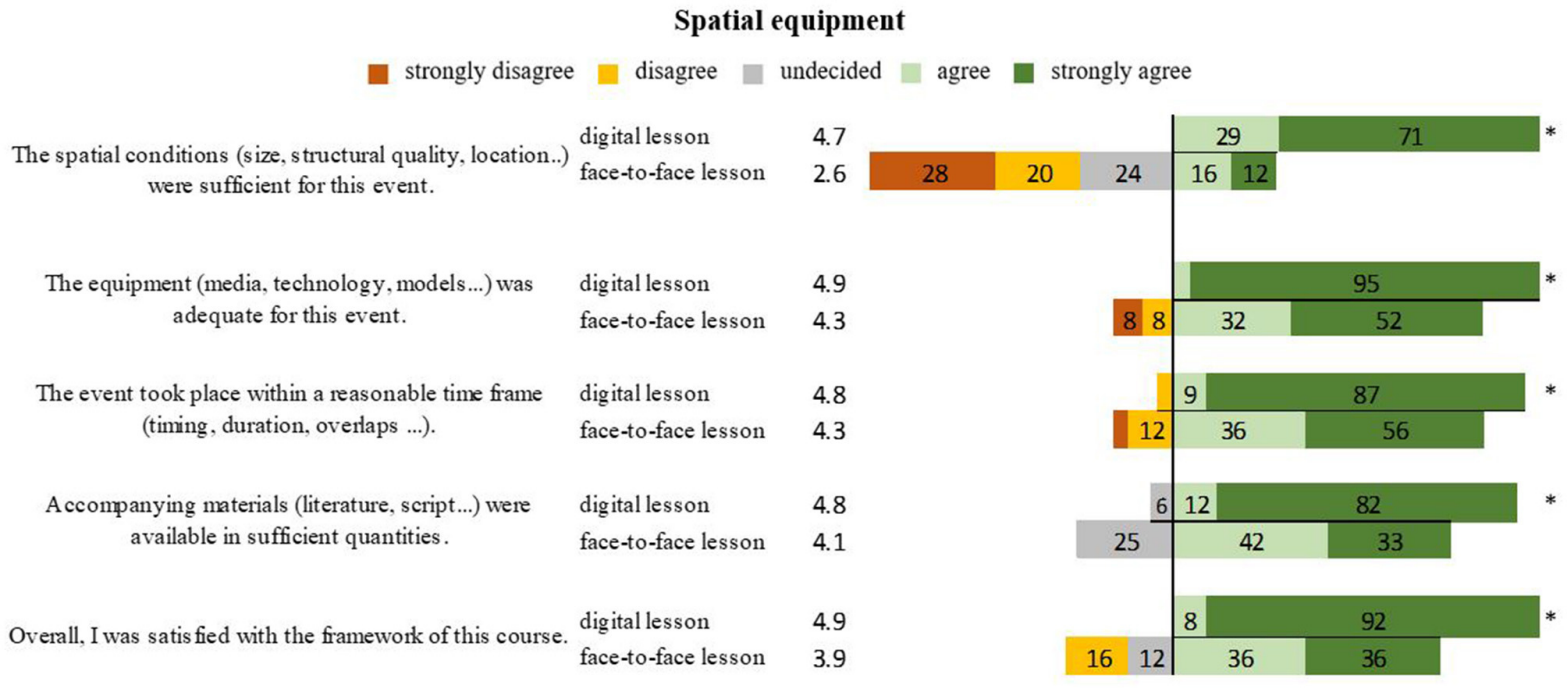
Representation of students' opinions on issues related to space in digital teaching and face-to-face teaching in percentages. *Marks significant differences.

Virtual teaching showed better ratings for all questions regarding spatial and equipment needs (spatial conditions *P* < 0.001, equipment *P* = 0.001, time frame *P* = 0.016, helpful accompanying materials *P* = 0.001). The overall rating of digital teaching was 4.9 and that of face-to-face instruction was 3.9 (*P* < 0.001) (Figure 1).

**Figure 2:**
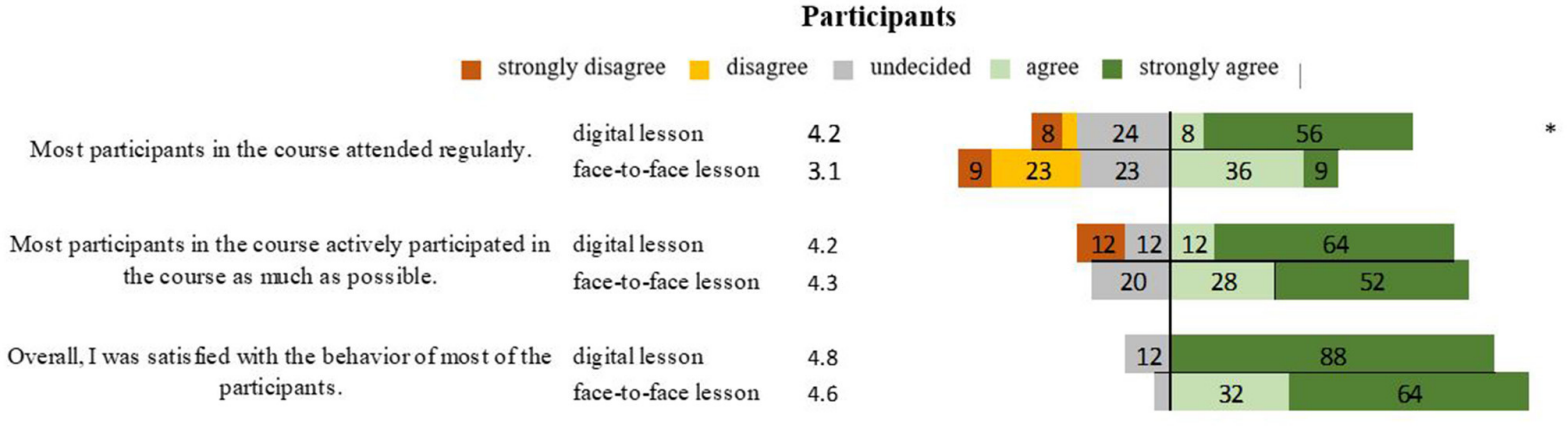
Representation of students' opinions of participation in digital and face-to-face teaching in percentages. *Marks significant differences.

The students rated that participation in virtual classes was better (*P* = 0.025) than it was for in-person participation (*P* = 0.939), and there was no significant difference in their overall behavior in digital (4.8) and in-person classes 4.6 (*P* = 0.180) (Figure 2).

**Figure 3:**
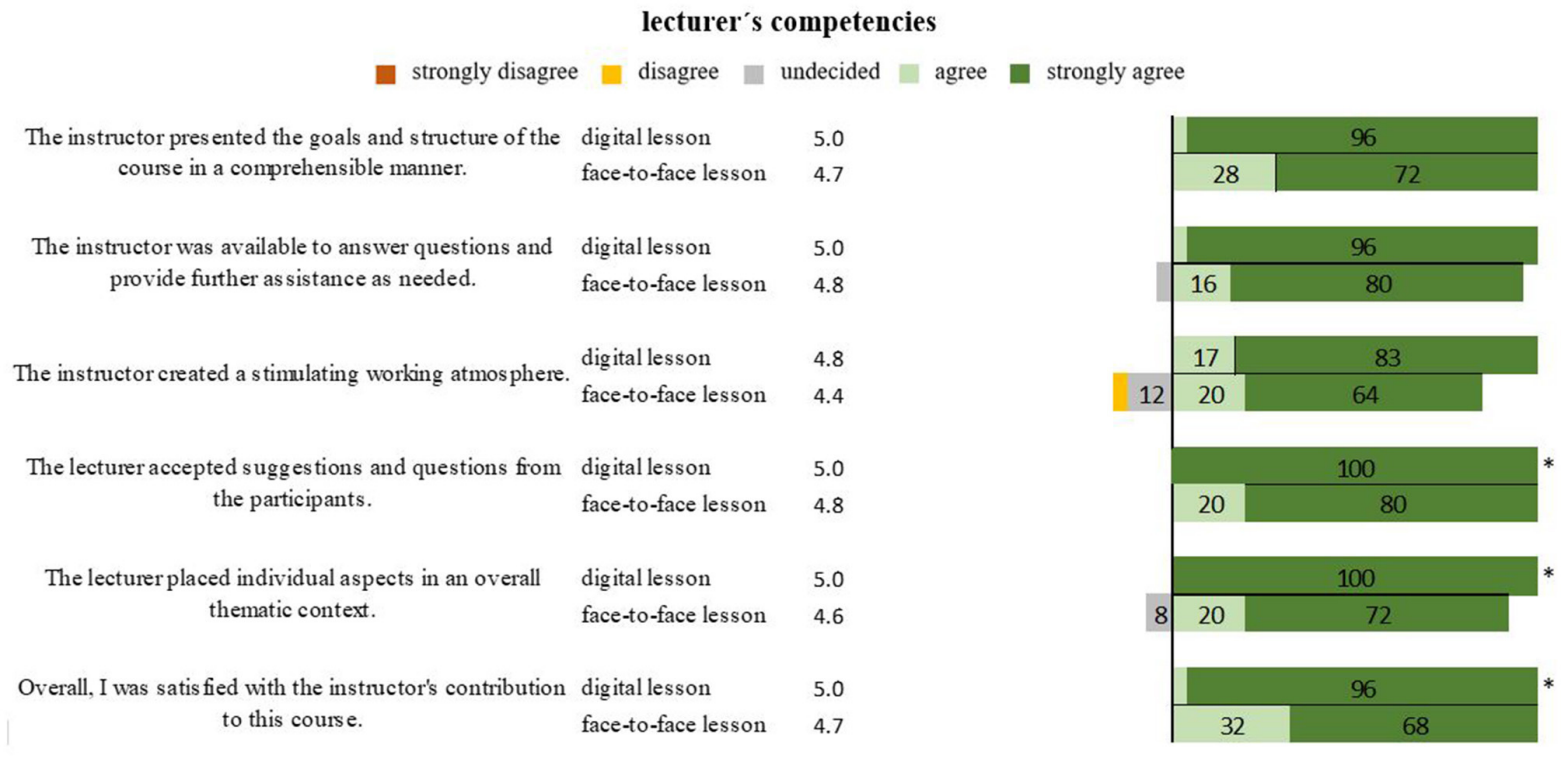
Representation of students' opinions of lecturers in digital and face-to-face teaching in percentages. *Marks significant differences.

There was no significant difference noticed by the students in lecturers' competencies in terms of communication of goals and structure (*P* = 0.052), thus allowing questions and assistance (*P* = 0.178) and creation of a stimulating working atmosphere (*P* = 0.104) in both lesson forms. In terms of taking up suggestions and questions regarding content (*P* = 0.017) and placing individual aspects in the overall context (*P* = 0.004), lecturers were significantly able to better demonstrate their competencies to the students in the virtual form of lessons than in the conventional form with an overall impression of instructor performance being 5.0 and 4.7 (*P* = 0.026), respectively (Figure 3).

**Figure 4:**
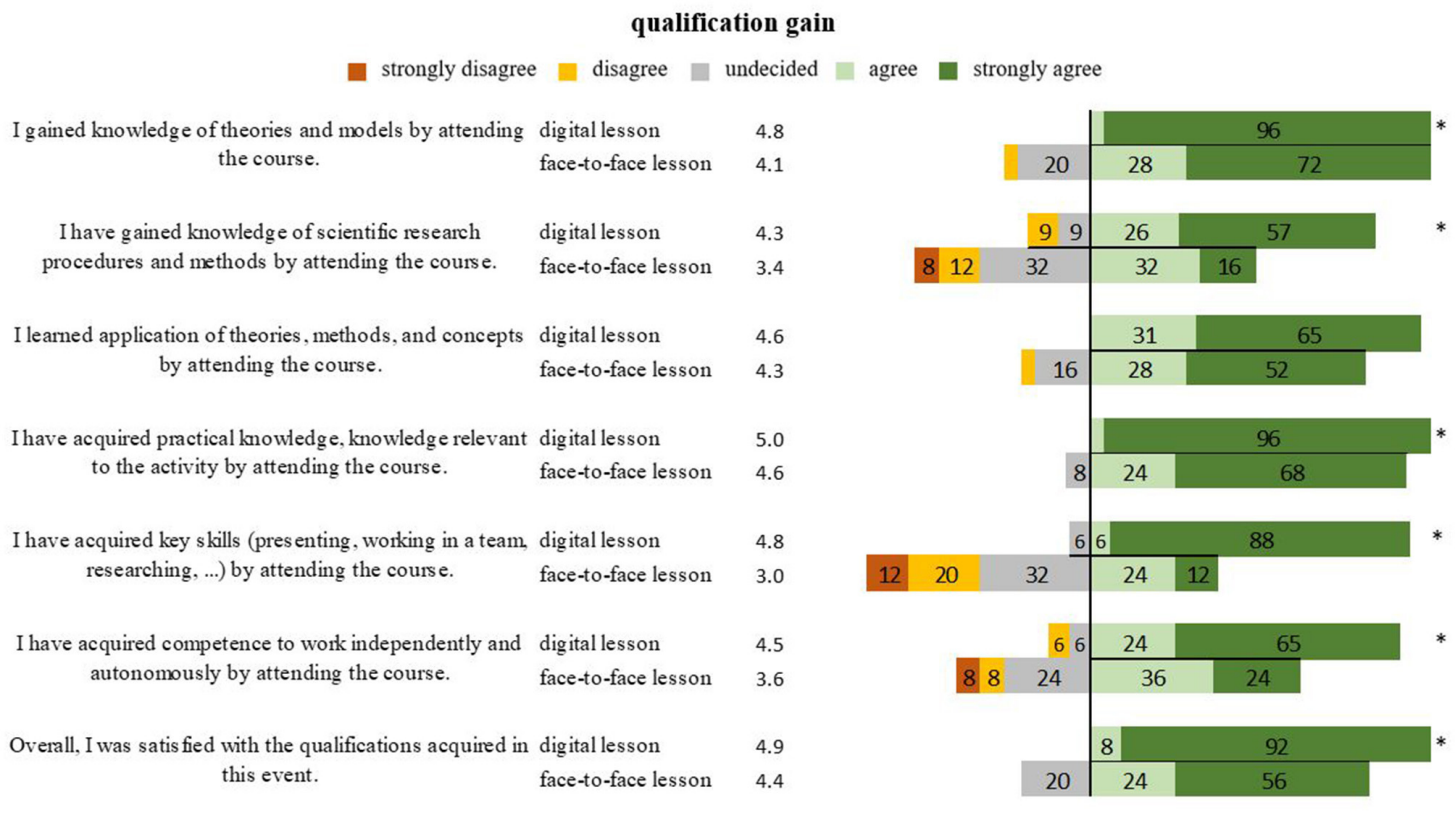
Representation of students' opinions on the skills and knowledge acquired in digital and face-to-face teaching in percentages. *Marks significant differences.

The qualification improvement with new knowledge (*P* = 0.001), research procedures (*P* = 0.005), practical knowledge (*P* = 0.023), the acquisition of key competencies (*P* < 0.001), and the competence of independent and autonomous working (*P* = 0.006) showed overall better ratings with 4.9 points for digital teaching and 4.4 (*P* = 0.005) for in-person teaching. The application of knowledge from both lesson forms was equally evaluated (*P* = 0.346) (Figure 4).

**Figure 5:**
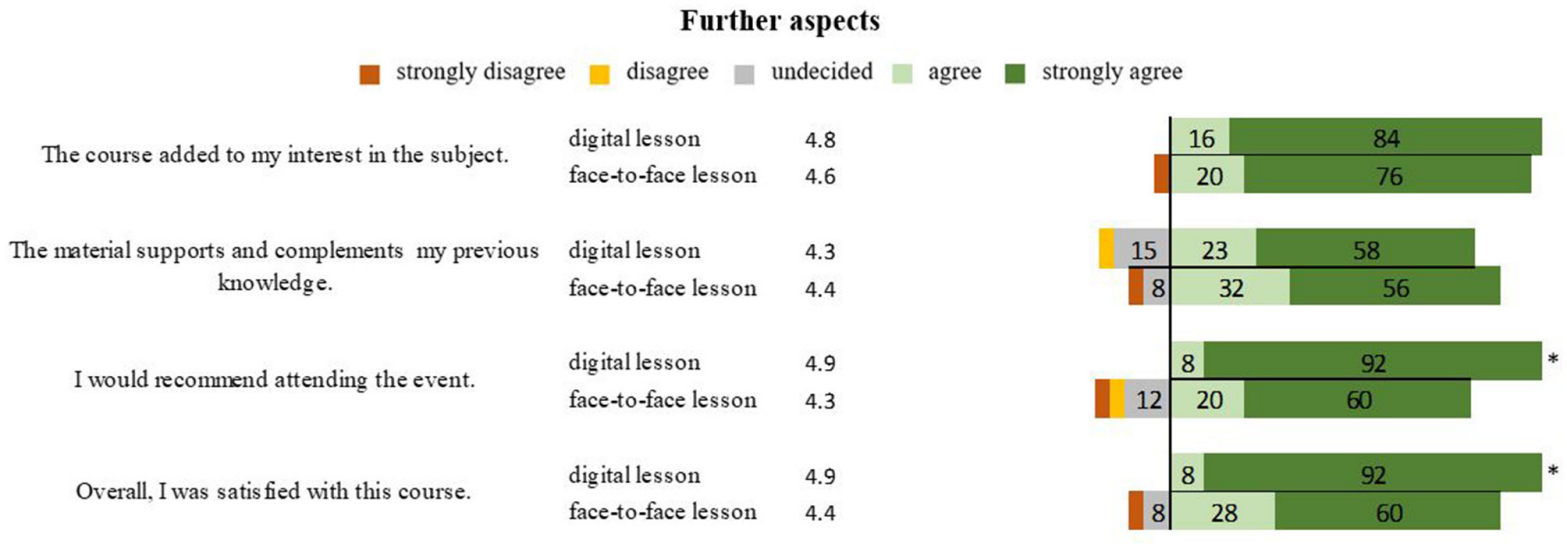
Presentation of students' opinions on the aspects of interesting and recommendable design in digital and face-to-face teaching in percentages. *Marks significant differences.

Further aspects such as awakening interest in the topic (*P* = 0.588) and linking to previous knowledge (*P* = 0.842) were equally evaluated in the lesson forms. The digital form was recommended to fellow students (*P* = 0.011), outweighing the conventional teaching, with the overall rating of 4.9 for the digital form being significantly better than 4.4 (*P* = 0.013) for conventional teaching (Figure 5).

The overall satisfaction with the course (4.9 vs. 4.4; *P* = 0.013) and the gain in skills (4.9 vs. 4.4; *P* = 0.005), and the satisfaction with the instructors (5.0 vs. 4.7; *P* = 0.026) were predominant for digital teaching. The overall satisfaction with the participants was equally evaluated for both forms (4.7 vs. 4.6; *P* = 0.596).

### Qualitative results

Table 2 visualizes the qualitative results, specifically the five themes with the most-relevant specified subthemes.

**Table 2:** Representation of the lecturers' opinions and qualitative results.

Framework	No time pressure Suitable equipment/less compatibility problems No cramped quarters Pleasant environment
Contact to students	Impersonal Inactive lecture format No network beyond lecture More distant formal atmosphere Performance oriented More concentrated
Own competencies	High practical part less teachable without practical applications Action–reaction missing Feedback missing New competencies through handling of technology New acquisition of techniques Time-consuming initiative
Knowledge transfer	Longer preparation time Lack of accessibility due to lack of feedback Little practical relevance of examination techniques/materials for visualization
Lecture format as lecturer	No direct contact with students and other lecturers Unpleasant due to unfamiliar structure Impersonal Higher pressure/pressure to perform to convey content Lower self-confidence

Within the 5 themes and specified subthemes.

#### Framework conditions

According to the instructors, a controversial picture emerged. The lecturers welcomed the advantages of time management without tiresome travel, the search for parking spaces, and the correct premises. At the same time, they favored their own rooms with a pleasant indoor climate. However, the deficits in the technical conditions of the conventional lecture with compatible equipment (laptop) to the university equipment (connections) also became apparent.

#### Contact with the students

Contact with the students was experienced as distant and impersonal in the digital lectures compared to conventional in-person events. Lively discussions on the topics did not arise. The lecturers experienced the students as performance-oriented and focused on the lecture. The usually accompanying networking after the lecture, for example as a search for doctoral theses or mentoring partners, was not possible in the digital form of teaching.

#### Lecturer's own competencies

When asked to self-reflect on the teaching given, the lecturers found it more difficult to convey the teaching, especially the lecture on sports medicine with high practical components as illustrative materials or demonstration of the examination techniques were not possible. The technical innovations required the lecturers to improve their computer situation both with hardware, software, and skills. The acquisition of new skills usually had to be done on one's own initiative and proved to be very time-consuming. All lecturers found it more difficult to assess their teaching success due to the lack of verbal and nonverbal feedback from the students.

#### Knowledge transfer

The lecturers found the digital lecture form to be more time-consuming for preparation to illustrate the practical parts of the lecture. In particular, the examination techniques could not be demonstrated or improved by practicing. The missing illustrative materials, such as bandaging options could not be demonstrated for better understanding. Overall, it remains more difficult for all the lecturers to assess what the students learned.

#### Lecturers' opinion on format

The main criticism of virtual lectures in the Sports Medicine course was the unfamiliar impersonal atmosphere. The lecturers found it not only more difficult to assess their teaching success but also more pressure to redesign the lecture for knowledge transfer. They felt diminished self-confidence to teach without student feedback.

## Discussion

COVID-19 is an ongoing pandemic and continues to impact everyday life (19). Due to the ongoing pandemic status, the digital *ad hoc* teaching formats established at the beginning of the COVID-19 pandemic have been established in everyday teaching in the last three pandemic years. Both the students and the lecturers have become accustomed to the new circumstances of knowledge transfer and digital teaching formats at medical universities as they are regularly established as a result (20). This can have advantages as well as disadvantages for both the students and the lecturers. With digital teaching formats, sick students can follow the teaching material from home and sick lecturers can teach from home without quarantine or other people (groups) being infected or endangered (20).

In addition to these advantages, there was also a permanent change compared to digital teaching concepts from the point of view of the students and lecturers. At the beginning of the pandemic, the establishment of digital teaching formats was often viewed negatively from the point of view of the lecturers since the implementation was a major challenge for the lecturers and the medical universities (21). At the beginning of the establishment of digital teaching concepts, the students had a positive attitude, although this attitude changed over the course of the pandemic years (22). The once divergent picture between lecturers and students is getting closer over the course of time. Lecturers were able to acquire digital skills and taught more digitally as students wanted to create more teaching formats in attendance. Therefore, the different reasons are known (23). As one of the main reasons, the explicit students of study by Olmes et al. named the poor quality of the teaching formats in digital form, the lack of human exchange with other students, or even the lecturers (24). In addition, it was complicated and difficult to convey practical teaching content to students through digital teaching formats. There were various reasons and the demand for newly created digital teaching concepts for this topic was great (25). To be able to establish new teaching concepts in the long term, it is important to know the attitude students and lecturers have toward digital teaching formats with complex and practical teaching contents (25). At this point, the present study analyzes the view of the students and lecturers on digital teaching formats and classroom teaching and examined which teaching concept can be applied to complex, practice-oriented teaching contents for the subject of sports medicine in both study groups.

A key advantage of digitization is the ability to automate processes. Many processes in teaching can be automated by digital solutions. These solutions speed up the processes and reduce the barriers from both the students' and lecturers' points of view, as well as reduce the costs of room rentals, travel routes, and energy costs. The biggest challenge is the transformation of classroom learning formats into digital learning formats. From the lecturers' point of view, there are no standardized courses of action for medical teaching at universities. Thus, the implementation of digitization depends or falls on the will and ability of the lecturers (26). As aforementioned in other studies and consistent with the results of the present study, digital formats are used in the longer term if they are user-friendly. This study served as a basis for this. The aim was to survey and verbalize the demands and attitudes of students and lecturers at a medical university during the *ad hoc* digitization process due to the COVID-19 pandemic. In this way, the present findings can contribute to improving the digitization processes for medical students and lecturers during the current semester. In addition to the positive effects of time management, the lecturers also expressed the reduction in long commuting distances within the clinical routine. This may have a positive effect on practicability; as even in the case of illness, teaching can be carried out by a substitute person or from home. This, in turn, has advantages for the students, as there is less teaching or they feel that they are a burden in the clinical routine of the lecturers. In addition, practical and complex teaching content can be taught using digital processes and manifested from the student's perspective. In the present study, for example, the students rated virtual teaching better for the preparation of active participation in attendance-based courses. The lecturers felt that the digital lecture format was more time-consuming in preparing and illustrating the practical parts of the lecture. In particular, the testing techniques could not be demonstrated or improved by practicing. The missing illustrative materials, such as bandages, could not be shown to better understand these possibilities. Overall, it is still more difficult for all lecturers to assess what the students have learned. Certainly, today's generation of students have grown up in the digital world, while the teachers have had to learn it (27). Sandars et al. (27) showed that students felt that their instructors were willing to improve their digital literacy skills and that some online teaching formats would persist after the COVID-19 pandemic (24). These were confirmed by Theoret and Ming, who found that online teaching and continued online communication could become pillars of medical education (28). Indeed, online teaching has been shown to promote self-learning, be as successful as traditional didactics, and provide an enjoyable experience for participants (29, 30). Overall, although most German medical educators were directly involved in patient care, and therefore, currently under significant stress, the COVID-19 pandemic has offered numerous opportunities for the use of digital media (31). Due to the uncertainty of the lecturers in dealing with digital media and the lack of time resources, the faculties must intervene in a supportive manner. There may be long-term changes due to the pandemic toward teaching using virtual media. Hopefully, the current wave of digitization will continue and the positive effects will persist.

### Limitations

The study is based on a survey study. Response bias can skew both student and faculty results. In addition, the maximum number of lecturers surveyed is limited to three. Therefore, the results are only of limited significance and do not reflect the views of lecturers from all the medical universities in Germany. This also applies only to the perspective of the surveyed students. These cannot be transferred uniformly to all students. In addition, the survey of the participants took place only at the University of Jena.

## Conclusion

The digital establishment of new tools and formats in teaching is not enough—they must also use sensible and planned basis so that they can also be used sustainably in the field of medical education. The basic prerequisite for the application is to know the attitude and the user needs of the students and lecturers. Regarding this purpose, this study was able to provide fundamental insights. In summary, it can be deduced that digital teaching is actively implemented by the lecturers and students due to the coronavirus pandemic. From a student perspective, digital formats could be used for the preparation and delivery of complex and practice-oriented teaching content. But from the lecturers' point of view, this requires more resources and requires the lecturers to have digital skills. In general, it can be observed that digital formats are in no way inferior to in-person teaching formats, as was assumed for a long time, but positively affects the quality of teaching.

## Data availability statement

The original contributions presented in the study are included in the article/supplementary material, further inquiries can be directed to the corresponding author.

## Author contributions

IG and SH conceived and planned the experiments. MK performed the analysis. FL, DB, and ES provided critical feedback and helped shape the research, analysis, and manuscript. All authors contributed to the article and approved the submitted version.
